# Prediction of celiac disease associated epitopes and motifs in a protein

**DOI:** 10.3389/fimmu.2023.1056101

**Published:** 2023-01-19

**Authors:** Ritu Tomer, Sumeet Patiyal, Anjali Dhall, Gajendra P. S. Raghava

**Affiliations:** Department of Computational Biology, Indraprastha Institute of Information Technology, New Delhi, India

**Keywords:** celiac disease, gluten immunogenic peptides, HLA-DQ2/DQ8, ensemble method, motif

## Abstract

**Introduction:**

Celiac disease (CD) is an autoimmune gastrointestinal disorder causes immune-mediated enteropathy against gluten. Gluten immunogenic peptides have the potential to trigger immune responses which leads to damage the small intestine. HLA-DQ2/DQ8 are major alleles that bind to epitope/antigenic region of gluten and induce celiac disease. There is a need to identify CD associated epitopes in protein-based foods and therapeutics.

**Methods:**

In this study, computational tools have been developed to predict CD associated epitopes and motifs. Dataset used for training, testing and evaluation contain experimentally validated CD associated and non-CD associate peptides. We perform positional analysis to identify the most significant position of an amino acid residue in the peptide and checked the frequency of HLA alleles. We also compute amino acid composition to develop machine learning based models. We also developed ensemble method that combines motif-based approach and machine learning based models.

**Results and Discussion:**

Our analysis support existing hypothesis that proline (P) and glutamine (Q) are highly abundant in CD associated peptides. A model based on density of P&Q in peptides has been developed for predicting CD associated peptides which achieve maximum AUROC 0.98 on independent data. We discovered motifs (e.g., QPF, QPQ, PYP) which occurs specifically in CD associated peptides. We also developed machine learning based models using peptide composition and achieved maximum AUROC 0.99. Finally, we developed ensemble method that combines motif-based approach and machine learning based models. The ensemble model-predict CD associated motifs with 100% accuracy on an independent dataset, not used for training. Finally, the best models and motifs has been integrated in a web server and standalone software package “CDpred”. We hope this server anticipate the scientific community for the prediction, designing and scanning of CD associated peptides as well as CD associated motifs in a protein/peptide sequence (https://webs.iiitd.edu.in/raghava/cdpred/).

## 1 Introduction

Celiac disease (CD) is an auto-immunological disorder which mainly affects the small intestine of the infected person (1). CD is a life-long disorder occurred due to the gluten associated foods which is found in various foods such as wheat, barley, spelt, kamut, and rye (2). The prevalence rate of CD is around 1.4% worldwide and it may vary with genetic and environmental factors. The occurrence of disease is significantly higher in children in comparison to adults (3). Various studies revealed that celiac disease patients develop inflammatory immune responses against gluten peptides. The innate immune responses cause toxic effects by gluten peptides on the intestinal epithelium due to increased production of cytokines such as interleukin-15 (4–7). However, the presence of certain class-II human leukocyte antigens (HLAs) molecules play a crucial role in the induction and regulation of immunological responses. The binding of gluten peptides with the HLA-DQ2/DQ8 receptors activates the adaptive immune responses (8). Whereas, HLA-DQ2 found in almost 94.5% of CD cases and HLA-DQ8 present in 2.7% of the cases (9). These binders are also linked with other autoimmunological disorders such as HLA-DQ8 associated with Type I diabetes (10).

**Figure 1:**
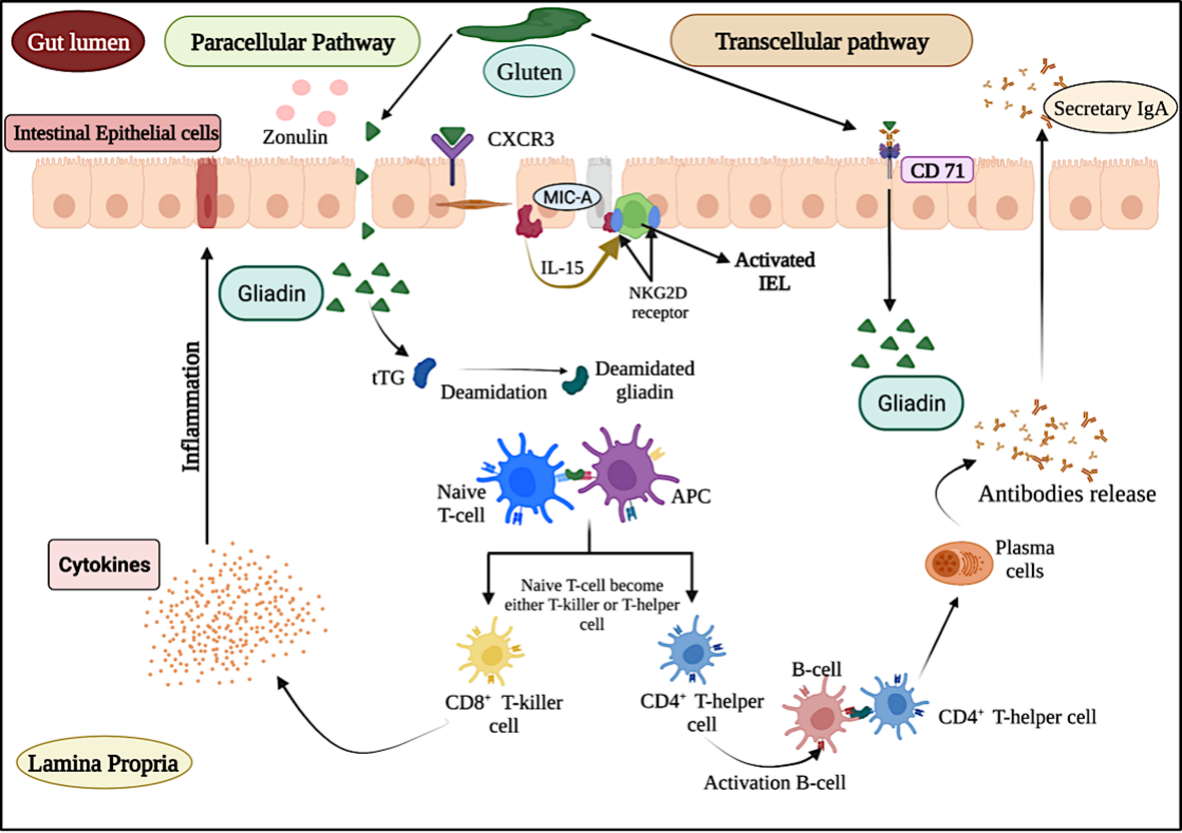
Schematic representation of celiac disease pathogenesis and immune response.

As depicted in **Figure 1**, the entry of gluten inside the lamina propria region of small intestine follows transcellular and paracellular pathways (11). In transcellular pathway, the entry of gluten is associated with the binding of secretory IgA (sIgA) in the apical region of intestine (12). However, in the paracellular pathway, the entry of gluten is associated with the binding of chemokine receptor 3 (CXCR3) present at enterocyte with the release of zonulin protein (13, 14). After entering inside the lamina propria region, a series of events trigger an inflammatory cascade which leads to the excessive release of antibodies (anti-tissue transglutaminase, anti-IgA antibodies and anti-endomysial antibodies) and cytokines (15) and ends with damage to the intestinal villi.

Due to auto-inflammatory immune responses several gastrointestinal disorders like malabsorption, vomiting, bloating, diarrhoea, abdominal pain and distension occurred (16). Recently, a number of biological and genetic tests (such as detection of antibodies, intestinal tissue biopsy, HLA-typing and gluten challenge test) are available for the disease detection (1). It has been found in many studies that α-gliadin 33-mer peptide having the property of resistant to gastrointestinal cleavage and makes it highly immunogenic peptide (5, 17–19). Despite tremendous understanding of CD, effective treatment for the disease is life-long gluten free diet. In order to manage severity of CD effectively, it is important to identify CD associated epitopes or immunogenic peptides responsible for CD. Identification of CD associated epitopes/peptides is not only important for identifying CD free food/therapeutic proteins, it is also important for designing antigen-based immunotherapy against CD.

In the pilot study, we have developed a computational approach for the prediction of CD associated peptides. We have extracted the experimentally validated CD-associated peptides from the IEDB database. In order to create negative dataset, we have collected CD non-causing peptides and random peptides from IEDB and Swiss-Prot, respectively. We have identified highly conserved regions of disease-causing peptides using motif-based search. In addition, we have developed prediction models using composition-based features and machine learning algorithms. In order to facilitate the community, we have provided the webserver and standalone package for the prediction and scanning of CD causing protein/peptides using sequence information.

## 2 Material and methods

**Figure 2:**
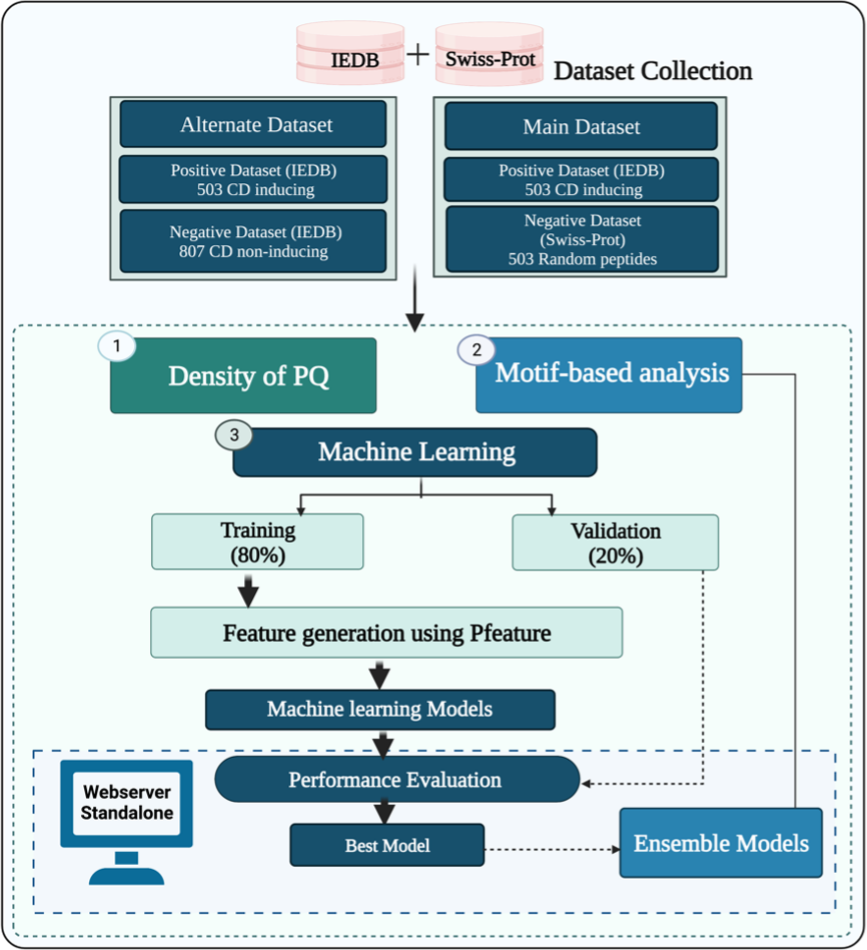
Overall architecture of the study.

The complete architecture of our study is illustrated in **Figure 2**. The detail of each step is described below.

### 2.1 Dataset collection and pre-processing

In this study, we have collected experimentally validated peptides from the immune epitope database (IEDB) (20). At first, we extracted a total of 521 unique celiac disease (CD) associated peptides from IEDB as a positive dataset. Further, we have selected unique peptides with a length of 9-20 amino-acid residues and got 503 CD associated peptides. Secondly, we extracted experimentally validated CD non-associated peptides from IEDB and random peptides from Swiss-Prot database (21). The main dataset incorporates 503 CD associated called positive peptides and 503 random peptides called negative peptides. The alternate dataset consists of 503 CD associated and 807 non-associated peptides (which can cause autoimmune disorders other than celiac disease). Finally, we obtained two datasets, i.e., the main dataset comprises an equal number of positive and negative peptides and alternate dataset 503 positive and 807 negative peptides.

### 2.2 Sequence logo

In order to understand the preference of amino-acid residues at a specific position, we have generated a one sample logo using WebLogo software (22). This tool needs a fixed length input sequence vector. Since, the minimum length of peptides in our datasets is 9 residues, so we have extracted 9-mers from N-terminal and 9-mers from C-terminal from each peptide. After that, we re-join both the regions in order to create a fixed length vector of 18 amino acids. The sequences of 18-residues were generated for all the peptides of both positive and negative datasets and used for the creation of one sample logo plots.

### 2.3 Amino-acid composition

We have used Pfeature (23) software for the computation of composition-based features. In this current study, we have computed amino acid composition based (AAC) features. In the case of AAC, the composition of each residue is computed in the peptide sequences and a vector od 20 length is generated using the equation 1.


[1] $$AAC_{i}=\frac{R_{i}}{L}\;\times \;100$$

Where, *AAC_i_* is amino-acid composition of residue type *i*, *R_i_* is the number of residues in *i*, and *L* is the length of peptide sequence.

### 2.4 Machine learning models

We have employed a number of machine learning algorithms for the classification of CD-causing peptides. Currently, we have used Scikit-learn (24) python library for the implementation of several classifiers including Decision Tree (DT), Random Forest (RF), XGBoost (XGB), Gaussian Naïve Bayes (GNB) Logistic Regression (LR), ExtraTree classifier (ET), and k-nearest neighbors (KNN).

### 2.5 Five-fold cross validation

In order to avoid overfitting, we have train, test and validate the machine learning models by employing five-fold cross validation technique as implemented in previous studies (25–28). At first, the complete dataset was divided into 80:20 ratio, where 80% dataset used for the training and 20% used for the external validation (29, 30). The five-fold cross-validation process is implemented on the 80% training dataset. In this process, the entire training dataset was divided into five equal sets, where each set is used for training and validation purpose. At first, four sets were used for training and fifth set was used for the testing, similarly the process is repeated five times so that each set can be used as testing dataset. Finally, we calculated the average performance of five sets which resulted after five iterations.

### 2.6 Model evaluation

In this study, we have used standard parameters for the evaluation of prediction models. Here, we have calculated both threshold dependent as well as independent parameters. In the case of threshold-dependent parameters we have computed, sensitivity (Sens), specificity (Spec), accuracy (Acc) and Matthews correlation coefficient (MCC) using the following equations (1-4). In addition, we have measured the performance of models with a well-established and threshold-independent parameter Area Under the Receiver Operating Characteristic (AUROC) curve.


[2] $$Sensitivity=\;\frac{T_{P}}{T_{P}+F_{N}}$$

[3] $$Specificity=\;\frac{T_{N}}{T_{N}+F_{P}}$$

[4] $$Accuracy=\;\frac{T_{P}+T_{N}}{T_{P}+T_{N}+F_{P}+F_{N}}$$

[5] $$F1-Score=\;\frac{2T_{P}}{2T_{P}+F_{P}+F_{N}}$$

[6] $$MCC=\;\frac{\left(T_{P}*T_{N}\right)-\left(F_{P}*F_{N}\right)}{\sqrt{\left(T_{P}+F_{P}\right)\left(T_{P}+F_{N}\right)\left(T_{N}+F_{P}\right)\left(T_{N}+F_{N}\right)}}$$

Where, T_P_, T_N_, F_P_ and F_N_ stand for true positive, true negative, false positive and false negative, respectively.

### 2.7 Ensemble method

The ensemble method is a hybrid approach in which both motifs based, and machine learning methods combined to achieve better performance. In this method, first motif-based approach is used to identify the disease-causing peptides and then we use machine learning methods to predict those peptides which are not covered by the motif-based approach. Finally, we generate an ensemble method which is a combination of both motif-based approach and machine learning method.

### 2.8 Web implementation

We have developed a webserver named “CDpred” for the prediction of CD associated peptides. The webserver is implemented by HTML5, JAVA, CSS3 and PHP scripts and compatible on several devices such as iMac, desktop, tablet and mobile. The webserver provides five user-friendly modules such as predict, PQ density, motif scan, protein scan, and design.

## 3 Results

### 3.1 Positional conservation analysis

**Figure 3:**
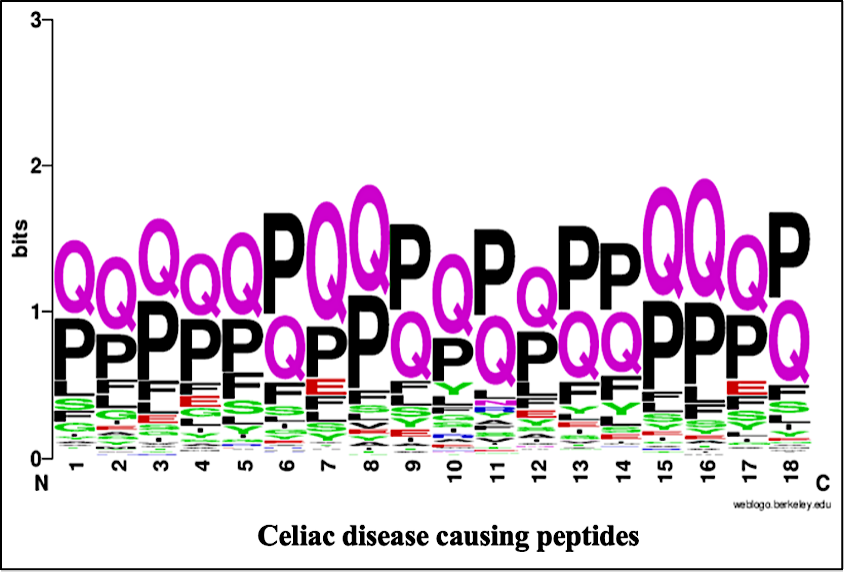
WebLogo of celiac disease-causing peptides.

The specific position of a residue is important for specific role and structure arrangement of a particular peptide or protein. To identify the most significant position of an amino acid residue in the peptide, we perform the positional analysis of CD causing peptides and CD non-causing peptides by using WebLogo (See **Figure 3**). It is worth noting that the first nine locations correspond to peptide N-terminal residues, whereas the latter nine positions correspond to peptide C-terminus. Here, we found that the proline (P) and glutamine (Q) residues are highly prominent at every position while the Phenylalanine (F) and glutamic acid (E) are also found at some positions.

### 3.2 Composition analysis

**Figure 4:**
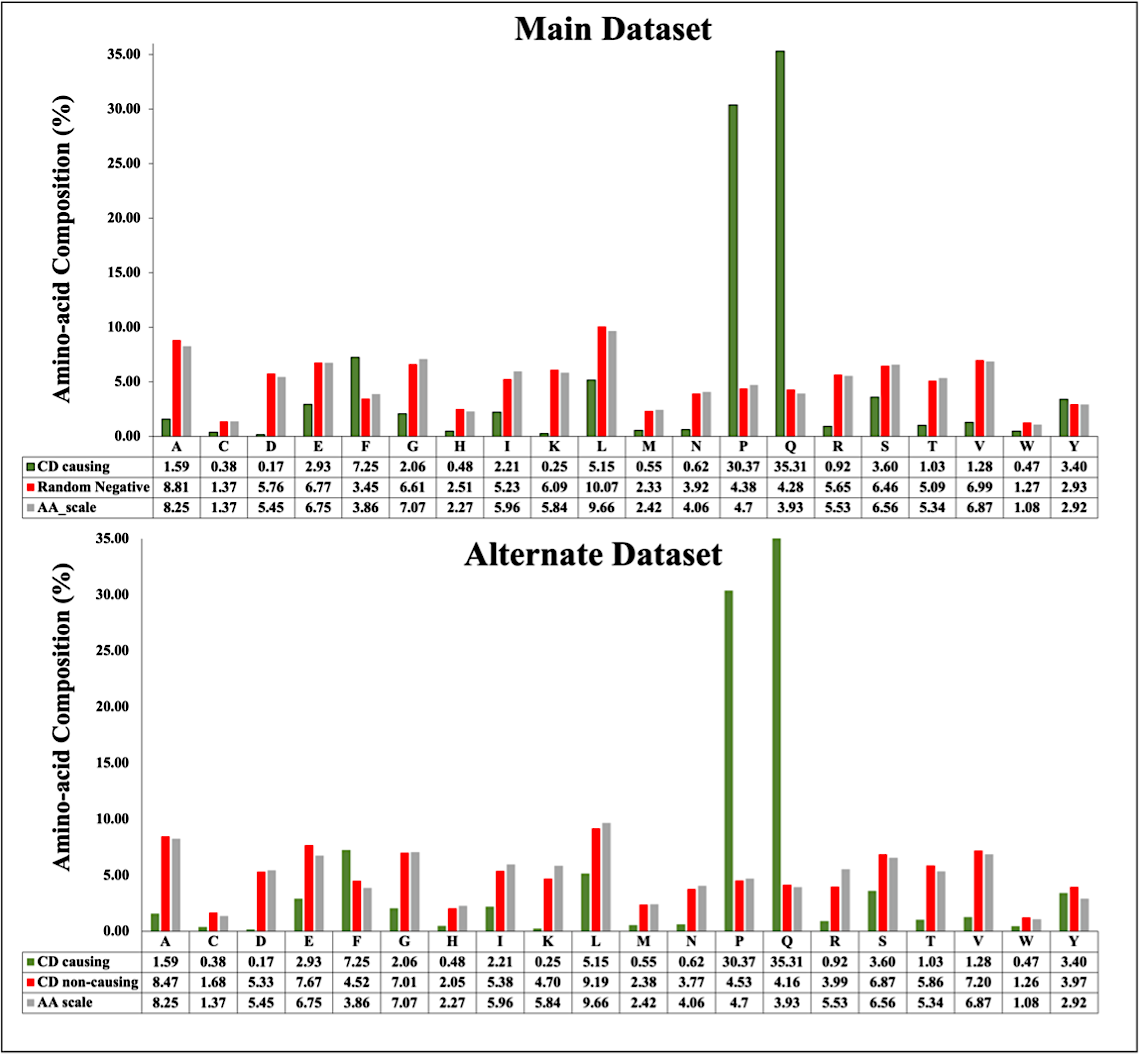
Average amino acid composition of peptides in main dataset and alternate dataset.

We compute the amino acid composition for main and alternate datasets. **Figure 4** depicts the average composition of CD inducing and non-inducing peptides. In CD causing peptides, the average composition of Proline (P), Glutamine (Q) and Phenylalanine (F) is higher in comparison with disease non-causing peptides, negative random and general proteome.

### 3.3 Frequency of HLA alleles

In the past, a number of studies report that celiac disease occurred due to the presence of certain HLA molecules such as HLA-DQ2 and HLA-DQ8 (29, 31, 32). Sallese et al. indicated that, in addition to HLA-DQ2 and HLA-DQ8, non-HLA variants are also associated to CD susceptibility (33). As shown in **Table 1**, we observed that maximum CD-associated peptides are HLA-DQ2/DQ8 binders, while some of the CD-associated peptides are linked with other HLA-alleles. This shows that innate (non-HLA-DQ mediated) and adaptive (HLA-DQ mediated) immune responses can be caused by gluten peptides. The complete frequency distribution of HLA-alleles binders of CD causing and non-causing peptides are given in **Supplementary Table S1**.

**Table 1:** Distribution of HLA alleles in CD causing and non-causing peptides.

HLA		CD causing (Positive)	CD non-causing (Negative)
HLA-class I	HLA-A	13	0
HLA-class II	HLA-DQ2	263	148
	HLA-DQ8	18	110
	HLA-DQ2/DQ8	24	9
	HLA-DR	3	402
	Other	182	138
	Total	503	807

### 3.4 Motif-based analysis

Motifs are known as the specific regions of a protein sequence which helps to identify the amino acid arrangement shared by a family of protein. The motifs are identified in the CD causing peptide sequences by MERCI program. The MERCI program helps to identify the motif regions in a set of sequences. We utilized the MERCI tool to look for motifs seen only in CD-causing peptides and not in disease non-causing or random peptides. We also looked for motifs found only in disease non-causing and random peptides. Here, we found 50 motifs in CD causing peptides of different length in which P and Q residues are present in abundance in CD causing peptides. We also checked the common motifs found in disease causing, non-causing and random negative peptides. The list of motifs and their occurrence in all the three datasets are given in **Table 2**.

**Table 2:** Abundance of motifs in CD-causing, non-causing and random negative peptides.

Motifs	Positive	Random Negative	Non-CD-causing
**QPF**	276	0	4
**QQPF**	170	0	1
**PYP**	120	0	3
**PEQ**	56	0	4
**QPQ**	350	1	0
**PQPQ**	189	1	0
**QQPQ**	131	1	0
**PQL**	84	1	0

### 3.5 PQ density

On performing the compositional and motif analysis, it was found that (P) and (Q) are the most abundant residues in CD-causing peptides as compared to non-causing peptides. In order to classify the peptides based on the PQ density, we have first generated the overlapping patterns of window size ranging from 3 to 9 for each peptide, since 9 was the minimum length of the peptides, and calculated the composition of residues P and Q in each pattern. Each peptide in the dataset is represented by the maximum value of composition for the respective pattern size and found the optimal composition at which we can classify the peptides with balanced sensitivity and specificity. To find the optimal pattern size, we have varied the size from 3 to 9, and found out that window size 5 and 6 performed best among the other sizes for main and alternate datasets, respectively as shown in **Table 3**.

**Table 3:** Performance of the PQ abundance-based method on different window sizes.

Main Data					
Window size	Threshold	Sensitivity	Specificity	Accuracy	AUROC
**3**	0.670	85.686	98.807	92.247	0.971
**4**	0.510	91.650	96.620	94.135	0.977
**5**	0.410	93.837	94.235	94.036	0.978
**6**	0.340	95.427	92.445	93.936	0.978
**7**	0.290	96.421	91.650	94.036	0.979
**8**	0.380	93.241	97.018	95.129	0.981
**9**	0.340	94.235	96.620	95.427	0.981
Alternate Data					
**Window size**	**Threshold**	**Sensitivity**	**Specificity**	**Accuracy**	**AUROC**
**3**	0.670	85.686	98.761	93.740	0.970
**4**	0.510	91.650	97.770	95.420	0.977
**5**	0.410	93.837	96.159	95.267	0.979
**6**	0.340	95.427	94.796	95.038	0.980
**7**	0.290	96.421	94.300	95.115	0.981
**8**	0.260	97.018	92.937	94.504	0.983
**9**	0.340	94.235	98.017	96.565	0.982

### 3.6 Machine learning based prediction

Various machine learning classifiers such as RF, DT, GNB, XGB, KNN, ETN, SVCN and LR are used to develop a prediction model. For this, we have computed the features of disease causing and disease non-causing peptides using composition-based module of Pfeature.

#### 3.6.1 Performance of AAC based features

Firstly, we have computed features of amino acid composition, using which we applied different machine learning techniques. As shown in **Table 4**, ET achieves maximum performance in comparison to other models with AUROC 0.991 and 0.995 and accuracy 96.02 and 97.03 on both training and validation dataset with a good balance of sensitivity and specificity in main data. Similarly, ET achieves maximum performance in comparison to other models with AUROC 0.995 and 0.999 and accuracy 97.519 and 98.092 on both training and validation dataset with a good balance of sensitivity and specificity in alternate data.

**Table 4:** The performance of machine learning classifiers on AAC based features on main and alternate dataset.

	Main dataset							
	Training				Validation			
Classifier	Sensitivity	Specificity	Accuracy	AUROC	Sensitivity	Specificity	Accuracy	AUROC
**DT**	92.269	92.556	92.413	0.962	97.059	91.000	94.059	0.982
**RF**	95.262	95.533	95.398	0.989	98.039	97.000	97.525	0.994
**LR**	96.010	96.030	96.020	0.988	98.039	96.000	97.030	0.990
**XGB**	95.761	95.782	95.771	0.987	98.039	93.000	95.545	0.995
**KNN**	95.262	95.285	95.274	0.986	97.059	96.000	96.535	0.991
**GNB**	93.017	98.263	95.647	0.976	93.137	98.000	95.545	0.990
**ET**	96.010	96.030	96.020	0.991	98.039	96.000	97.030	0.995
**SVC**	95.761	95.782	95.771	0.987	97.059	96.000	96.535	0.991
	Alternate dataset							
	Training				Validation			
**DT**	92.537	92.570	92.557	0.968	94.059	99.379	97.328	0.990
**RF**	97.015	97.368	97.233	0.995	98.020	97.516	97.710	0.998
**LR**	96.269	96.285	96.279	0.990	97.030	96.273	96.565	0.987
**XGB**	97.015	97.059	97.042	0.992	99.010	93.168	95.420	0.998
**KNN**	95.771	95.975	95.897	0.992	98.020	95.652	96.565	0.995
**GNB**	92.537	97.059	95.324	0.977	96.040	96.273	96.183	0.983
**ET**	97.512	97.523	97.519	0.995	98.020	98.137	98.092	0.999
**SVC**	97.015	96.904	96.947	0.993	98.020	96.894	97.328	0.996

# DT, Decision tress; RF, Random Forest; LR, Logistic regression; XGB, XGBoost; KNN, k-nearest neighbour; GNB, Gaussian naïve base; ET, Extra tree classifier; SVC, support vector classifier.

#### 3.6.2 Performance of ensemble model

In ensemble method, first we used the motif-based approach by identifying the coverage of motifs in the given protein/peptide sequences. Our motif-based approach achieves 81.71% accuracy in the independent dataset as shown in **Table 5**. The rest sequences, which were not predicted using motif-based approach, were covered by using the machine learning method. By combining both approaches we achieve the highest performance of 100% accuracy on independent dataset. Our ensemble method is the best approach for predicting the CD associated peptides.

**Table 5:** The table shows the occurrence of motif in positive sequences with their cumulative coverage.

Motif	Occurrence	Percentage	Cumulative
**QPF**	276	54.87	54.87
**PQQP**	41	8.15	63.02
**PYP**	33	6.56	69.58
**QPQQ**	28	5.57	75.15
**PFP**	14	2.78	77.93
**PEQ**	12	2.39	80.32
**FPQP**	4	0.8	81.11
**FPQQ**	2	0.4	81.51
**PQLP**	1	0.2	81.71
**ML Prediction**	92	18.29	100

### 3.7 Services to scientific community

We design a user-friendly prediction web server that incorporates several modules to determine CD-causing peptides in order to serve the scientific community. The prediction models used in the study are implemented in the web server. Based on the prediction models’ score at a different threshold, users can predict whether a query peptide causes CD or not. The web server comprises five major modules 1) Prediction, 2) PQ Density, 3), Motif 4) Scan and 5) Design. The user can classify CD-causing peptides from disease non-causing peptides using the ‘Predict’ module. The “PQ Density” module used to calculate PQ content in a given query sequence based upon the window size. Users can map or scan CD-causing motifs in the query sequence using the “Motif” module. We used the MERCI software to extract themes from CD-causing peptides that had been empirically confirmed. The “Scan” module was used to scan the amino-acid sequence for CD-causing areas. The user can generate all potential analogs of the input sequence using the “Design” module. The positive and negative datasets utilized in this work are also available for download, and the peptide sequence are available in FASTA format. HTML, JAVA, and PHP scripts were used to create the web server CDpred https://webs.iiitd.edu.in/raghava/cdpred/. The server is user-friendly and compatible with a variety of devices, including computers, Android phones, iPhones, and iPads. In addition, we provided a standalone package in the form of a Docker container.

## 4 Case study: Evaluation of CDpred on external dataset

In this study, we evaluate our model by utilizing a new dataset (i.e., CD-associated peptide sequences) obtained from AllergenOnline database (http://www.allergenonline.org) (34) under celiac disease section (35). A total of 1040 unique experimentally validated CD-associated peptide were collected from AllergenOnline. We found 265 common sequences with our dataset (used for developing CDpred), hence we removed those sequences and left with 775 unique CD-associated new sequences. After that, we evaluate the performance of CDpred on independent dataset of 775 peptides using default parameters of “Ensemble module” and achieved 100% accuracy. Our method predicts all 775 CD-associated peptides correctly, where 661 peptides were predicted using Motif based approach and 114 using machine learning based approach.

## 5 Discussion and conclusion

Celiac disease is a chronic, genetically predisposed enteropathy triggered by gluten showing a wide spectrum of clinical manifestations (5, 29). It can be associated to a number of diseases such as cirrhosis, autoimmune hepatitis, diabetes mellitus, gluten ataxia, peripheral neuropathies, etc (36, 37). Moreover, CD is not limited to gastrointestinal tract, in fact it is associated with a number of extra-intestinal manifestations and other autoimmune disorders (38–41). The origins of the onset and appearance of related diseases may vary; for example, type 1 diabetes mellitus (T1D), share a common genetic basis; while others may have similar pathogenic pathways. Granito et al., also showed a significant correlation between anti-microfilament IgA and severity of intestinal damage in CD patients (42). Recent studies reported that, celiac patients have an elevated risk of getting small bowel cancer and intestinal lymphomas (43, 44).

Some autoimmune neurological manifestations including cerebral ataxia, peripheral neuropathy, epilepsy, dementia, and depression are associated with CD (45–49). Volta et al., found that there is a significant correlation between anti-ganglioside antibodies and neurological disorders in CD patients (50). Cervio et al., observed that antigliadin and anti-tTG antibodies in CD patients are linked to the development of neurological disorders (51). Moreover, immune-related diseases are also occurred due to gluten intake for example, atopy (52). The only effective lifelong treatment of this disease is a gluten-free diet. Due to increased number of cases in worldwide a number of gluten-free products are available for celiac susceptible people (18, 53). Thus, it is essential to identify or eliminate gluten immunogenic peptides from the food products which can induce the celiac disease and sensitive to celiac patients.

In this study, we have made a systematic attempt for the prediction of peptides responsible for causing the disease. We have collected the dataset from IEDB and Swiss-Prot databases. We have created two datasets for the analysis and prediction of CD causing peptides. The positive dataset contain experimentally validated peptides obtained from IEDB that are responsible for celiac disease. These peptides are not only gluten peptides (high frequency of P & Q) but also associated with celiac disease.

In addition, we have created alternate dataset where we have taken negative set contain peptides which cause diseases other than celiac disease. This is not necessary that all the gluten peptides are responsible for the celiac disease. There are few gluten peptides which showed toxic effect on intestinal epithelium cells and induce innate immune response (4). *L. Maiuri et al.* also showed the effect of gluten peptides on mucosal surface of the celiac patients and healthy individuals (54, 55). In this study, we observed that amino acid residues (P and Q) are highly abundant in CD causing peptides in comparison with negative and random peptides. The similar findings are supported by the previous studies where they have shown the abundance of P and Q amino acids in gluten proteins (56, 57). From the motif-based approach we identified certain motifs (QPQ, QPF, PQPQ, QQPF, QQPQ, PYP), which are highly conserved in CD causing peptides in comparison with CD non-causing peptides. So, we performed PQ based analysis where we calculate the abundance of PQ residues in the CD causing and non-causing peptides. In addition, we have developed prediction models using amino-acid composition-based features. We achieved maximum performance with AUROC of 0.99 on the training and validation datasets, respectively. We have also developed an ensemble method by combining both motif-based approach and machine learning based models. This ensemble approach provides the 100% accuracy on independent dataset. In addition, we have developed a webserver named CDpred (https://webs.iiitd.edu.in/raghava/cdpred/), standalone package (https://webs.iiitd.edu.in/raghava/cdpred/standalone.php) and GitLab (https://gitlab.com/raghavalab/cdpred) for the prediction of CD causing peptides.

## Data availability statement

The original contributions presented in the study are included in the article/**Supplementary Material**. Further inquiries can be directed to the corresponding author.

## Author contributions

RT, AD and GR collected and processed the datasets. RT, SP and GPSR implemented the algorithms and developed the prediction models. RT, AD, SP and GR analysed the results. RT and SP created the back-end of the web server the front-end user interface. RT, AD, and GR penned the manuscript. GPSR conceived and coordinated the project. All authors contributed to the article and approved the submitted version.
