# Supplementary material for: Prediction of celiac disease associated epitopes and motifs in a protein
Source: Front Immunol. 2023 Jan 19;14:1056101. doi: 10.3389/fimmu.2023.1056101 (PMC9893285; doi:10.3389/fimmu.2023.1056101)
Supplement: Supplementary file 1 [file Table_1.docx]

**Table S1: Distribution of HLA alleles in CD causing and non-causing peptides**

| **HLA** | **Haplotype/Allele** | | **CD -causing** | **Other autoimmune disorders** |
| --- | --- | --- | --- | --- |
| **HLA class I** | **HLA-A** | HLA-A*01:01 | 2 | 0 |
|  |  | HLA-A*02:01 | 1 | 0 |
|  |  | HLA-A*08:01 | 8 | 0 |
|  |  | HLA- class I | 2 | 0 |
| **HLA class II** | **HLA-DQ2** | HLA-DQ2 | 110 | 28 |
|  |  | HLA-DQA1*02:01/DQB1*02:01 | 3 | 2 |
|  |  | HLA-DQA1*02:01/DQB1*02:02 | 6 | 4 |
|  |  | HLA-DQA1*05:01/DQB1*02:01 | 122 | 47 |
|  |  | HLA-DQB1*02:01 | 2 | 67 |
|  |  | HLA-DQB1*03:02 | 20 | 0 |
|  | **HLA-DQ8** | HLA-DQ8 | 9 | 9 |
|  |  | HLA-DQA1*03:01/DQB1*03:01 | 3 | 4 |
|  |  | HLA-DQA1*03:01/DQB1*03:02 | 6 | 97 |
|  | **HLA-DQ2/DQ8** | HLA-DQA1*03:01/DQB1*02:01 | 6 | 0 |
|  |  | HLA-DQA1*05:01/DQB1*03:02 | 18 | 9 |
|  | **Other** | HLA-class II | 57 | 0 |
|  |  | HLA-DQ | 5 | 114 |
|  |  | HLA-DQA1*02:01/DQB1*03:03 | 1 | 0 |
|  |  | HLA-DQA1*03:02/DQB1*03:03 | 2 | 2 |
|  |  | HLA-DR3 | 1 | 117 |
|  |  | HLA-DR4 | 1 | 206 |
|  |  | HLA-DR7 | 1 | 79 |
|  |  | HLA-DR | 0 | 0 |
|  |  | NA | 117 | 22 |
| Total | | | 503 | 807 |
